# The role of latency reversal in HIV cure strategies

**DOI:** 10.1111/jmp.12613

**Published:** 2022-08-27

**Authors:** Kiho Tanaka, Youry Kim, Michael Roche, Sharon R. Lewin

**Affiliations:** ^1^ Department of Infectious Diseases The University of Melbourne at The Peter Doherty Institute for Infection and Immunity Melbourne Victoria Australia; ^2^ Victorian Infectious Diseases Service Royal Melbourne Hospital at The Peter Doherty Institute for Infection and Immunity Melbourne Victoria Australia; ^3^ Department of Infectious Diseases Alfred Hospital and Monash University Melbourne Victoria Australia

**Keywords:** HIV cure strategies, HIV, HIV reservoir, immunotherapy, immune checkpoint blocker, latency reversal

## Abstract

One strategy to eliminate latently infected cells that persist in people with HIV on antiretroviral therapy is to activate virus transcription and virus production to induce virus or immune‐mediated cell death. This is called latency reversal. Despite clear activity of multiple latency reversal agents in vitro, clinical trials of latency‐reversing agents have not shown significant reduction in latently infected cells. We review new insights into the biology of HIV latency and discuss novel approaches to enhance the efficacy of latency reversal agents.

## 1 INTRODUCTION

Antiretroviral therapy (ART) is unable to cure HIV infection due to the persistence of a reservoir of long lived and proliferating latently infected cells. HIV latency occurs when the virus is integrated into the host cell DNA but does not produce viral proteins or virions, and therefore, the infected cells are not visible to immune‐mediated clearance. One strategy to eliminate or reduce the pool of latently infected cells is termed shock and kill, where the latent provirus is activated leading to immune‐mediated clearance or death through viral cytolysis (reviewed in^1^). A large number of latency‐reversing agents (LRAs) have been identified, many of which can reverse HIV latency in vitro, ex vivo and in animal models.^1^, ^2^ Despite this, in clinical trials in people with HIV (PWH) on ART, some but not all LRAs have been shown to induce virus transcription and/or virion production, but there has been minimal or no reduction in the reservoir.^2^ An increase in virus transcription has been demonstrated in human clinical trials of histone deacetylase inhibitors (HDACi),^3^, ^4^, ^5^ the PKC agonist bryostatin‐1,^6^ the toll‐like receptor‐9 (TLR) agonist Lefitolimod^7^ and immune checkpoint inhibitors.^8^, ^9^, ^10^ There remains a need to enhance our understanding of the biology of the latent reservoir as well as develop novel LRAs that are more potent, more specific and can also induce cell death.

## 2 NEW INSIGHTS INTO FACTORS THAT CONTROL HIV LATENCY

### 2.1 Varying HIV transcriptional activity in T‐cell subsets

HIV DNA can be found in multiple CD4^+^ T‐cell subsets with recent evidence demonstrating that genetically intact HIV proviruses were more common and more likely to persist over time in effector memory CD4^+^ T cells compared to naive, central and transitional memory CD4^+^ T cells.^11^ More mature T‐cell subsets also have a higher level of basal virus transcription^12^, ^13^ and commonly used LRAs appear to have different levels of potency in different T‐cell subsets, with memory stem cells from PWH on ART being highly resistant to HIV activation.^12^, ^14^ Latently infected cells from blood can also vary in their responsiveness to activation, with some infected cells requiring multiple stimuli to reactivate the provirus; however, this feature was not related to the site of integration.^15^

Viral transcriptional activity also differs between blood and tissue sites and may reflect the different cellular makeup of specific tissues, such as lymph nodes or the gastrointestinal tract.^16^, ^17^, ^18^, ^19^ Recent evidence has shown that there are multiple blocks to completion of HIV transcription in latently infected cells, with specific blocks to transcriptional elongation and splicing.^20^ Furthermore, these blocks to completion of transcription differ between latently infected cells isolated from blood and tissue.^18^ It appears that most LRAs can induce initiation of viral transcription but are unable to overcome blocks to elongation and splicing, therefore limiting the potential for virion production and therefore cell death.^21^

### 2.2 Site of HIV integration influences transcriptional activity

Basal and inducible HIV transcriptional activity in latently infected cells has been recently shown to be partially controlled by the site of provirus integration.^22^ Using a new technique of parallel HIV RNA, integration site and proviral sequencing (PRIP‐Seq) which can analyse single cells for integration site, viral sequence and viral RNA, the levels of basal transcription from intact virus in CD4^+^ T cells from PWH on ART were shown to decline on ART, raising the possibility that over time the reservoir is enriched for more deeply latent viruses.^22^ Using the same technique and cells from elite controllers (PWH who can naturally control HIV replication in the absence of ART), intact proviruses were preferentially found in transcriptionally inaccessible sites such as centromeric satellite DNA and sites with heterochromatin features,^23^ suggesting that in both elite controllers and long‐term ART, there is selection for a less transcriptionally active reservoir.

### 2.3 Extrinsic factors influencing HIV transcription

**FIGURE 1:**
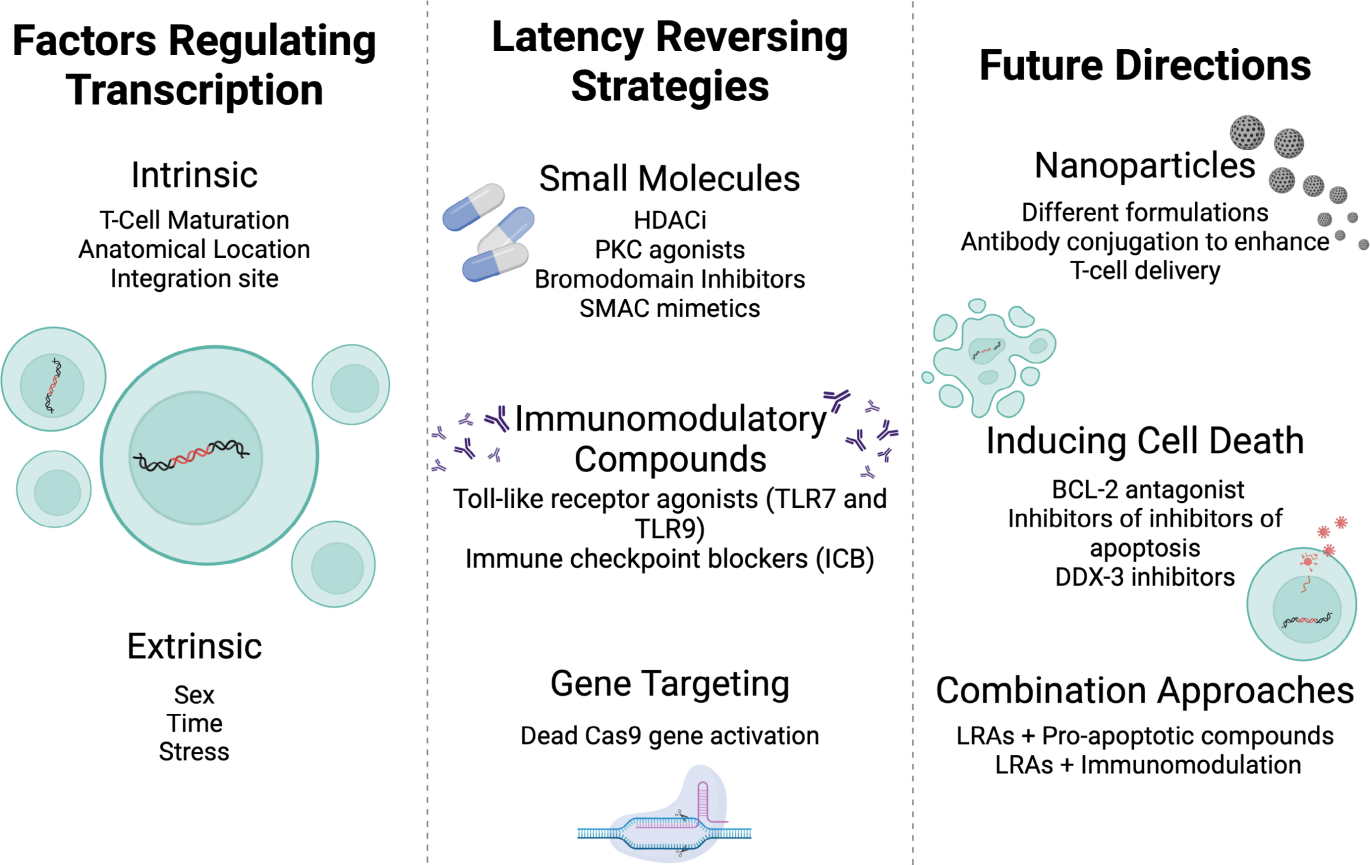
Factors modulating HIV transcription on ART and strategies to reverse HIV latency. Both intrinsic and extrinsic factors regulate HIV transcription within latently infected CD4^+^ T cells that persist in people with HIV on antiretroviral therapy. Understanding each of these factors will identify new targets to reverse HIV latency. Latency reversal has been demonstrated with small molecules (including histone deacetylase inhibitors (HDACis), bromodomain inhibitors and protein kinase C (PKC) agonists); immunomodulatory compounds (including toll‐like receptor agonists (TLR7 and TLR9) and immune checkpoint blockers (ICB) and gene targeting (using dead Cas9 gene activation)). Future directions aimed at enhancing the potency and specificity of latency reversal include nanoparticle delivery, the induction of cell death and ultimately a combination of these approaches

In addition to cellular factors that determine basal levels of HIV transcription, other extrinsic factors can impact viral transcription, including sex, time and stress. Women with HIV on ART have lower levels of plasma viraemia and cell‐associated multiply spliced HIV RNA compared to men.^24^ This observation may potentially be explained by higher levels of the oestrogen receptor (ESR)‐1 in women which has been shown to repress proviral activation.^25^ Our group has recently demonstrated that cell‐associated unspliced HIV RNA (which largely reflects evidence of transcription initiation) in PWH on ART varied temporally with a circadian rhythm.^26^, ^27^ This is likely through regulation of HIV transcription by the circadian transcription factors, circadian‐locomotor‐output‐cycles‐kaput (CLOCK) and brain‐and‐muscle‐ARNT‐like‐1 (BMAL1), which can bind to the E‐box in the HIV long terminal repeat (LTR).^27^, ^28^ We and others have also shown that psychological stress may also modulate viral transcription.^27^, ^29^, ^30^ Taken together, these findings demonstrate the complex multifactorial control of HIV transcription in HIV reservoirs (summarised in Figure 1) but at the same time has identified multiple new targets that could be exploited to enhance latency reversal.

## 3 ENHANCING POTENCY, FUNCTION AND SPECIFICITY OF LRAS

One key concern in relation to current LRAs is that they lack specificity as well as potency. Therefore, there is a large effort to identify novel targets, use immunomodulatory LRAs that have dual activities and/or increase the targeted delivery of these compounds.

### 3.1 Enhancing potency

Second mitochondria‐derived activator of caspases mimetics (SMACm) are a new class of LRA that can activate the HIV LTR via the non‐canonical nuclear factor kappa B (NFkB) pathway.^31^, ^32^ SMACm can also inhibit inhibitors of apoptosis and are being actively pursued as treatments for cancer.^33^ A large number of SMACm have been shown to potently reverse HIV latency in vitro using cell lines^31^, ^34^, ^35^ and two SMACm, AZD5582 and Ciapavir potently activated HIV latency in vivo using HIV‐infected mice and SIV‐infected non‐human primates (NHP).^34^, ^35^ There are currently no data on the activity of SMACm in vivo in PWH and it is unclear if off target effects of SMACm such as Bell's Palsy seen in the cancer setting^36^ will be a barrier to their use in the setting of HIV cure.

### 3.2 Enhancing function

Immunomodulatory LRAs have the benefit of dual function—reversing HIV latency and also enhancing HIV‐specific immunity. Immune checkpoint (IC) inhibitor molecules such as programmed cell death protein 1 (PD‐1), Lymphocyte activation gene 3 (LAG‐3) and T‐cell immunoreceptor with Ig and ITIM domains (TIGIT) are expressed on CD4^+^ T cells that are enriched for HIV latency.^37^, ^38^ Antibodies to immune checkpoints, either alone or in combination, have also been shown to reverse HIV latency using both in vitro models and patient‐derived cells.^38^, ^39^, ^40^ Blockade of PD‐1 with the anti‐PD‐1 antibody pembrolizumab was recently shown in a clinical trial to induce expression of both cell‐associated unspliced HIV RNA and plasma RNA in PWH on ART.^41^ Antibodies to immune checkpoints can also increase HIV‐specific T‐cell function ex vivo^42^ and in vivo;^43^ however, whether this increase in HIV‐specific T‐cell function can also clear infected cells in vivo remains unknown. Given the commonly reported immune‐related adverse events following anti‐PD‐1, that can be irreversible, there are some concerns about whether these antibodies can be safely pursued in PWH.^44^ Furthermore, a recent description of deleterious outcomes using anti‐PD‐1 and a therapeutic vaccine in an SIV‐infected non‐human primate animal model highlights the additional need for caution.^45^

Agonism of TLRs have also been shown to induce latency reversal and can enhance innate immune function.^46^, ^47^, ^48^, ^49^ TLR‐7 and TLR‐9 agonists induce latency reversal via stimulating type I interferon release and interferon‐stimulated genes without causing global immune activation.^46^, ^47^, ^48^, ^49^ TLR agonists combined with either broadly neutralising antibodies or a therapeutic vaccine induced a delay to viral rebound following cessation of ART in NHPs infected with SIV containing an HIV envelope (SHIV) and treated with ART during both acute infection and chronic infection.^50^, ^51^, ^52^ Recently, combining both active and passive immunisation with a TLR‐7 agonist induced virological control in 70% of SHIV‐infected non‐human primates following cessation of ART.^53^ Whether similar levels of virological control off ART can be achieved in PWH with similar combinations of interventions, remains to be determined. Importantly, the TLR‐7 agonist vesatolimod in PWH on ART was recently shown to be safe^54^ and also reduce intact proviruses and modestly delay viral rebound after cessation of ART.^55^ These studies provide the necessary data to now evaluate a TLR‐7 agonist in combination with other interventions. It is important to highlight that despite supportive in vitro data, it is unclear whether TLR agonists truly reverse HIV latency in vivo or whether their additional beneficial activity observed in NHP studies was related to activation of innate immune function.

### 3.3 Increasing specificity

Nanoparticles provide a novel pathway to enhance the specificity and potency of LRAs. Nanoparticle formulations loaded with LRAs including the protein kinase C agonist Bryostatin‐2 and the histone deacetylase inhibitor suberoylanilide hydroxamic acid have been shown to increase potency of latency reversal combined with targeted delivery to T cells.^56^, ^57^ Furthermore, lipid coated polynanoparticles that were loaded with two LRAs, Ingenol‐3A (Ing3A) and JQ1, induced synergistic effects on latency reversal.^58^ Conjugation of anti‐CD4 monoclonal antibody to the nanoparticle also resulted in the successful delivery of drug to lymph nodes, when administered by the subcutaneous route.^58^ Other strategies to target resting T cells to enhance the potency and specificity of LRAs include modification of size, charge and antibody conjugation (reviewed in^59^). Another approach to enhance the specificity of HIV latency reversal can be provided through Clustered Regularly Interspaced Short Palindromic Repeats (CRISPR) technology, such as the use of dead Cas9 activation to selectively bind and activate the HIV LTR.^60^, ^61^

## 4 STRATEGIES TO ENHANCE NON‐IMMUNE‐MEDIATED CELL DEATH

An emerging concept in HIV latency is that infected cells are primed for survival and therefore targeting this survival mechanism will facilitate selective death of infected cells. Factors that regulate apoptosis such as proteins from the B‐cell lymphoma (BCL)‐2 family and inhibitors of apoptosis proteins (IAPs) have been shown to be over‐expressed in latently infected CD4^+^ T cells.^62^, ^63^, ^64^, ^65^, ^66^ Therefore, there is high interest in targeting these specific proteins, including the use of the BCL‐2 antagonist, Venetoclax, which can increase selective death of latently infected cells ex vivo.^66^, ^67^ The IAP protein BIRC‐5 has been shown to be over‐expressed in latently infected cells,^64^ and inhibitors of this protein using either YM115^64^ or DDX3 inhibitors^68^ can induce death of infected cells ex vivo. In addition, SMACm which are inhibitors of IAP can induce autophagy‐dependent apoptosis in infected cells.^62^, ^63^ Finally, the triple combination of latency reversal (using bryostatin or anti‐CD3/anti‐CD28) and HIV‐specific CD8^+^ T cells with venetoclax reduced intact and inducible proviruses ex vivo using cells from PWH on ART.^66^

## 5 CONCLUSION

Our understanding of the complexity of HIV latency is rapidly expanding. The level of HIV transcriptional activity on ART is influenced by the HIV integration site, the differentiation status of the T‐cell and its anatomical location, as well as extrinsic factors such as sex, time and stress. New approaches that combine latency reversal and enhancement of immunity show some promise in animal models; however, the success achieved to date is yet to be replicated in human clinical trials. There is now high interest in the combination of LRAs with pro‐apoptotic agents, to specifically enhance elimination of latently infected cells. In conclusion, it is highly unlikely that latency reversal alone will eliminate the reservoir; however, this approach is critical to reduce the pool of infected cells and remains a core part of HIV cure strategies.

## ACKNOWLEDGEMENT

Open access publishing facilitated by The University of Melbourne, as part of the Wiley ‐ The University of Melbourne agreement via the Council of Australian University Librarians.

## FUNDING INFORMATION

SRL is an NHMRC practitioner fellow and is supported by the National Institutes of Health (NIH) Delaney AIDS Research Enterprise (DARE UM1 AI164560‐01).

## CONFLICT OF INTEREST

SRL's institution has received funding from the National Health and Medical Research Council (NHMRC) of Australia, National Institutes for Health, Wellcome Trust American Foundation for AIDS Research; Merck, ViiV and Gilead for investigator‐initiated research; Merck, ViiV and Gilead for educational activities. She is on the advisory boards of Vaxxinity, Immunocore, Gilead and Abbvie.

## Data Availability

Data sharing not applicable to this article as no datasets were generated or analysed during the current study.
